# Purulent pericarditis caused by methicillin-sensitive *Staphylococcus aureus* bacteriuria

**DOI:** 10.1186/s12872-024-03828-9

**Published:** 2024-03-13

**Authors:** Lorraine Mascarenhas, Dzhalal Agakishiev, Morgan Freeman, Scott Hubers

**Affiliations:** 1 Department of Medicine, University of Minnesota Medical School, Minneapolis, MN USA; 2 Gastroenterology Division, University of Minnesota, Minneapolis, MN USA; 3 Cardiology Division, Veterans Affairs Medical Center, Minneapolis, MN USA

**Keywords:** Purulent pericarditis, *Staphylococcus aureus* Bacteriuria, Asymptomatic bacteriuria

## Abstract

**Background:**

Purulent pericarditis (PP)— a purulent infection involving the pericardial space—requires a high index of suspicion for diagnosis as it often lacks characteristic signs of pericarditis and carries a mortality rate as high as 40% even with treatment. Common risk factors include immunosuppression, diabetes mellitus, thoracic surgery, malignancy, and uremia. Most reported cases of PP occur in individuals with predisposing risk factors, such as immunosuppression, and result from more commonly observed preceding infections, such as pneumonia, osteomyelitis, and meningitis. We report a case of PP due to asymptomatic bacteriuria in a previously immunocompetent individual on a short course of high-dose steroids.

**Case presentation:**

An 81-year-old male presented for severe epigastric pain that worsened with inspiration. He had been on high-dose prednisone for presumed inflammatory hip pain. History was notable for urinary retention requiring intermittent self-catheterization and asymptomatic bacteriuria and urinary tract infections due to methicillin-sensitive *Staphylococcus aureus* (MSSA). During the index admission he was found to have a moderate pericardial effusion. Pericardial fluid cultures grew MSSA that had an identical antibiogram to that of the urine cultures. A diagnosis of purulent pericarditis was made.

**Conclusion:**

PP requires a high index of suspicion, especially in hosts with atypical risk factors. This is the second case of PP occurring as a result of asymptomatic MSSA bacteriuria. Through reporting this case we hope to highlight the importance of early recognition of PP and the clinical implications of asymptomatic MSSA bacteriuria in the setting of urinary instrumentation and steroid use.

## Background

Purulent pericarditis (PP) is a localized infection of the pericardial space characterized by macroscopic or microscopic purulence [1]. The diagnosis once was a common complication of pneumococcal pneumonia, however, after the emergence of antibiotics it has become increasingly rare and comprises 1% of all pericardial disease cases [2, 3]. In addition to its rarity, the diagnosis requires a high degree of suspicion as ‘classic’ signs of pericarditis such as chest pain, pericardial friction rub, and diagnostic electrocardiogram (ECG) abnormalities, are often absent. If left untreated, PP carries a mortality rate of up to 100% [4–6]. Rarely occurring as a primary disease, PP more commonly develops through direct extension of intrathoracic infections or hematologic spread [7]. *Staphylococcus aureus* is the most common causative pathogen, but *Streptococcus* species, gram-negative organisms, *Mycobacterium tuberculosis*, and anaerobic bacteria have all been observed [6, 8]. Immunosuppression, diabetes, thoracic surgery, malignancy, and uremia are common risk factors for the disease, which typically presents with fever, tachycardia, and chest pain [4, 9]. The definitive diagnosis is established through echocardiography and pericardial fluid analysis, with treatment being antimicrobial therapy and pericardial fluid drainage often via pericardiocentesis or subxiphoid pericardiotomy [10]. Even with treatment, patients with PP have poor outcomes with mortality rates as high as 40% [11]. In these cases, death often occurs as a result of cardiac tamponade, constrictive pericarditis, or systemic toxicity [8]. The vast majority of reported cases have occurred in individuals with predisposing risk factors, such as immunosuppression, with most preceding infections being pneumonia, osteomyelitis, meningitis, otitis media, and skin infections [5]. We present an interesting case of PP due to methicillin-sensitive *Staphylococcus aureus* (MSSA) bacteriuria in the setting of frequent urinary instrumentation and high-dose steroid use. To our knowledge, there are only a few documented cases of PP occurring secondary to a urinary infection, of which only one case was due to MSSA bacteriuria [12–16].

## Case presentation

An 81-year-old male presented to our Emergency Department with severe, sharp, non-radiating, epigastric pain that worsened with inspiration. Past medical history included open-angle glaucoma, sciatica, urinary retention due to benign prostatic hyperplasia for which he self-catheterized twice daily, and urinary tract infections and asymptomatic bacteriuria due to MSSA resistant to trimethoprim-sulfamethoxazole. Two years prior to admission he presented for dysuria and received antibiotic treatment for a urinary tract infection due to this specific isolate of MSSA. The episode of asymptomatic bacteriuria occurred a few months prior to presentation when he underwent pre-operative workup for a cystolithotripsy for two bladder stones. Immediately prior to that procedure he received vancomycin as a pre-operative antibiotic. He tolerated the procedure well and did not experience any urinary tract infections after that.

He lived an active lifestyle with frequent exercise and denied worsening chest pain or dyspnea with exertion. Review of systems was negative for orthopnea, paroxysmal nocturnal dyspnea, and lower extremity edema. Notably, in the preceding two weeks of admission, he had several Emergency Department visits for left hip pain. A hip x-ray was negative for fracture, joint dislocation, and significant degenerative disease. There were no signs of joint erythema or effusion to warrant workup for septic arthritis. He was prescribed diclofenac gel, lidocaine patches, acetaminophen and, notably, a course of prednisone for presumed inflammatory hip pain. For 5 days prior to admission, he had been taking 60 milligrams of prednisone daily, with plans for a 12-day taper.

**Fig. 1:**
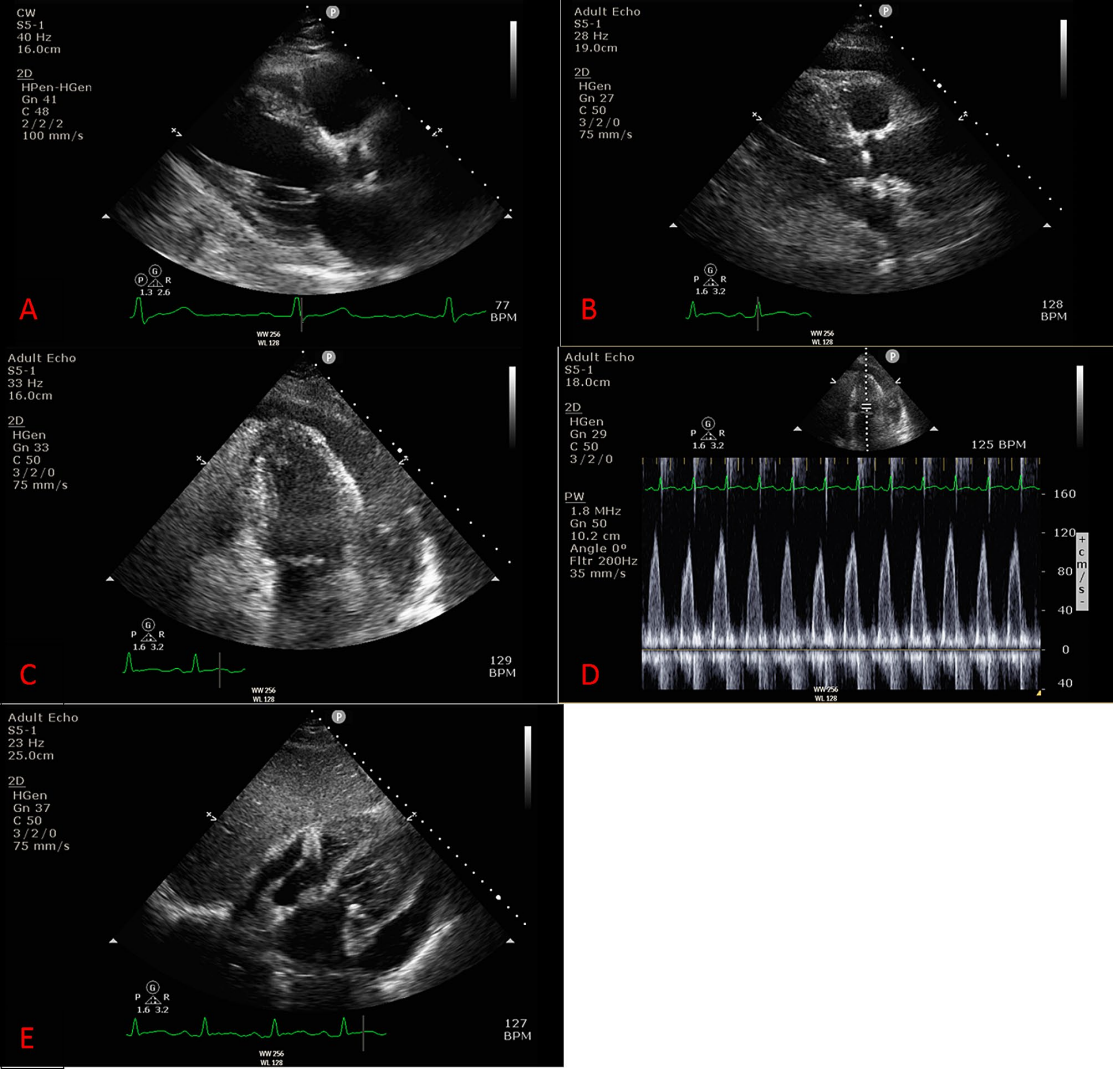
Echocardiographic Images Transthoracic echocardiogram on hospital day #1 showing no significant pericardial effusion on parasternal long axis (**A**). Repeat echocardiogram on hospital day #3 showing a large pericardial effusion on parasternal long axis (**B**) and apical (**C**) views. Mitral inflow variability on pulsed wave Doppler was present (**D**) along with right atrial wall collapse (**E**), concerning for a hemodynamically significant effusion

On presentation the patient was afebrile and hypertensive with a blood pressure of 168/81 mmHg. Labs were notable for a white blood cell count of 22.95 × 10^9^/L (4.0–11.0 × 10^9^/L), absolute neutrophil count of 21.55 × 10^9^/L ​​(2.0-7.7 × 10^9^/L), troponin of 0.174 ng/mL (≤ 0.028 ng/mL), with a repeat value at 0.165 ng/mL, and a C-reactive protein of 64.28 mg/L (≤ 5.0 mg/L). Urinalysis was notable for turbid urine with 2 + blood, 19 red blood cells per high power field, 49 white blood cells per high power field, and a leukocyte esterase of 250 leukocytes/µL. ECG revealed normal sinus rhythm without ST segment or T wave abnormalities. Chest x-ray demonstrated mild cardiomegaly, but was otherwise unremarkable, and an echocardiogram revealed normal biventricular function, an ejection fraction of 60–65%, and no evidence of regional wall motion abnormalities, valvular vegetations, or significant pericardial effusion (Fig. 1A).

The patient was admitted, and the next day underwent a nuclear stress test which did not expose any fixed or reversible perfusion defects. Later in the day, the patient developed severe back pain and tachycardia of 110 beats per minute. Computed tomography scan displayed a large pericardial effusion measuring 18 millimeters in thickness and bibasilar pulmonary atelectasis. Over the course of the day, the patient became febrile to 101.5º F, persistently tachycardic, and required 3 L per minute of oxygen via nasal cannula to maintain an oxygen saturation over 90%. The patient was started empirically on intravenous vancomycin and piperacillin-tazobactam after cultures of the blood, urine, and sputum were obtained. Inflammatory markers such as white blood cell count, C-reactive protein, erythrocyte sedimentation rate, and procalcitonin progressively increased. Repeat imaging was obtained and a chest x-ray demonstrated interval enlargement of the cardiac silhouette and worsening bibasilar opacifications. A second echocardiogram was performed and demonstrated a circumferential pericardial effusion measuring 2.5 centimeters to 3.0 centimeters with findings suggestive of evolving cardiac tamponade based on the mitral and tricuspid valve inflow variabilities, dilated inferior vena cava with reduced collapse on inspiration, subtle right atrial collapse, and sinus tachycardia (Fig. 1B-E).

The patient underwent pericardiocentesis during which 360 milliliters of red-brown, turbid pericardial fluid was removed. A pericardial drain was placed, and fluid was sent for analysis. Blood, urine, and pericardial fluid cultures grew MSSA resistant to trimethoprim-sulfamethoxazole, and > 100,000 colony forming units of this isolate of MSSA were present in the urine (**Table 1**).

**Table 1:** Antibiotic susceptibilities

*Staphylococcus Aureus* Cultures		Antibiotic Susceptibilities				
		Oxacillin	Trimethoprim-Sulfamethoxazole	Tetracyclines	Rifampin	Vancomycin
Pericardial Fluid^†^		S	R	S	S	S
Blood^†^		S	R	S	S	S
Urine*	9/30/2021^†^	S	R	S	S	S
	5/9/2021	S	R	S	S	S
	7/5/2020	S	R	S	S	S

*All urine cultures contained > 100,000 colony forming units

^†^Index hospitalization cultures

For 24 h while cultures finalized the patient was treated with intravenous vancomycin and cefazolin because of a positive nasopharyngeal polymerase chain reaction test for methicillin-resistant *Staphylococcus aureus* and clinical decompensation. Treatment was subsequently narrowed to intravenous cefazolin thereafter. Unfortunately, the patient became increasingly encephalopathic and pulled out the pericardial drain, after which he became progressively dyspneic and went into cardiac arrest with pulseless electrical activity. Cardiopulmonary resuscitation was initiated immediately and return of spontaneous circulation was achieved within 10 min. Repeat echocardiography revealed a recurrent moderate-sized effusion measuring 1.5 centimeters to 2.0 centimeters and signs of hemodynamic effect. The patient underwent a subxiphoid pericardiotomy with a pericardial ‘window’. Although there was no discrete source of bleeding, the patient was noted to have oozing and diffuse inflammation of the pericardial surface. A total of 200 milliliters of thin sanguineous fluid were removed during the procedure, and 2 chest tubes were placed. The patient was initiated on the post-arrest therapeutic hypothermia protocol (targeted body temperature of 33º Celsius) after which he was noted to have large amounts of sanguineous chest tube output. After discussion with the family, the patient was transitioned to comfort care measures and expired.

## Discussion

To our knowledge, this is the second documented case of PP occurring secondary to MSSA bacteriuria with subsequent bacteremia. Our patient was noted to have a history of urinary tract infections and asymptomatic bacteriuria due to MSSA resistant to trimethoprim-sulfamethoxazole—the same pathogen found in his blood, urine, and pericardial fluid during the index hospitalization. Although his bacteriuria was asymptomatic around the index hospitalization, we believe his self-catheterization led to mucosal trauma, as evident by hematuria on urinalysis, and his high-dose prednisone use created a degree of immunosuppression that predisposed him to hematogenous spread of the urinary organism. Furthermore, based on the history obtained, the patient did not have any new wounds, intravenous drug use, acupuncture treatment, recent trauma, or recent surgery, apart from the cystolithotripsy four months prior to presentation, to explain his MSSA bacteremia.

S. aureus is an uncommon isolate in the urine and has an incidence of 0.13–1% in all urine cultures and 0.5–6% in positive urine cultures. S. aureus bacteriuria (SABU) can present as asymptomatic bacteriuria or urinary tract infections, and common risk factors include indwelling urinary catheters, prior urinary tract instrumentation, urinary obstruction, long-term care, older age, hospital exposure, and malignancy [17, 18]. It can occur from hematogenous seeding of the urinary tract or serve as the primary nidus of infection with potential to cause bacteremia and invasive infections [17, 18]. S. aureus bacteremia (SAB) resulting from SABU has been previously studied. In a cohort study of 102 patients with SABU, 21% of patients developed SAB (13% within 4 days of bacteriuria and 8% within 12 months) [19]. Additionally, in a retrospective analysis of 132 patients with significant SABU (defined as > 10^5^ CFU/mL), 8.3% of patients developed bacteremia secondary to bacteriuria with blood cultures demonstrating identical antibiograms and phage types as the urine cultures [20]. Although SAB may lead to SABU, this has only been observed in a small proportion of patients (2.5%) without risk factors, such as indwelling urinary catheters [21].

Current guidelines recommend limiting treatment of asymptomatic bacteriuria to pregnant individuals and those undergoing endourological procedures associated with mucosal trauma [22]. Due to a lack of randomized controlled trials, there is no recommendation on screening for and treating asymptomatic bacteriuria in the setting of intermittent self-catheterization. Although our patient did not undergo an endourological procedure immediately prior to presentation, he likely had a degree of mucosal trauma from intermittently self-catheterizing, as evident by hematuria on urinalysis. While this alone may not be an indication for antibiotic treatment, this patient may have also had a degree of immunosuppression from his high-dose steroid use. Several studies suggest that prednisone doses over 5 milligrams per day for one or more weeks can increase the risk for serious bacterial infections due to immunocompromise and this risk is dose and duration dependent [23–27]. Therefore, because our patient was using 60 milligrams of prednisone daily in the setting of traumatic urinary instrumentation, we believe he was at higher risk for hematogenous spread of his urinary pathogen.

There are limitations to this report. First, it was based on a case report, therefore, generalizability is limited. Second, given the retrospective and observational nature of the case report no causal relationship between bacteriuria and purulent pericarditis can be made. Third, while we suspect the MSSA isolated in the urine and pericardial fluid are the same based on the susceptibility testing, we did not have whole genome sequencing capabilities to strengthen this argument and cannot definitively draw this conclusion.

Nonetheless, our presented case is unique given that our patient developed purulent pericarditis from short-term high-dose steroid use, intermittent self-catheterization, and SABU. Through reporting this case we aim to emphasize the potential clinical implications of steroid use in the setting of urinary instrumentation and asymptomatic bacteriuria. Future randomized controlled trials are necessary, however, to explore the risk of systemic infections in individuals who intermittently self-catheterize and have asymptomatic bacteriuria.

## Data Availability

The data analyzed for this case report is included in this published article, tables, and figures.

## Abbreviations

PP: Purulent pericarditis
ECG: Electrocardiogram
MSSA: Methicillin-sensitive *Staphylococcus aureus*
CT: Computed tomography
UTI: Urinary tract infection
SABU: *Staphylococcus aureus* bacteriuria
SAB: *Staphylococcus aureus* bacteremia
